# Bushen Huoxue Recipe Alleviates Implantation Loss in Mice by Enhancing Estrogen–Progesterone Signals and Promoting Decidual Angiogenesis Through FGF2 During Early Pregnancy

**DOI:** 10.3389/fphar.2018.00437

**Published:** 2018-05-15

**Authors:** Jiahui Ding, Xiujuan Tan, Kunkun Song, Wenwen Ma, Jing Xiao, Yufan Song, Mingmin Zhang

**Affiliations:** Institute of Integrated Traditional Chinese and Western Medicine, Tongji Hospital, Tongji Medical College, Huazhong University of Science and Technology, Wuhan, China

**Keywords:** Bushen Huoxue recipe, implantation loss, estrogen, progesterone, decidual angiogenesis, FGF2, traditional Chinese medicine, early pregnancy loss

## Abstract

Bushen Huoxue recipe (BSHXR) is a classic Chinese herbal prescription for nourishing the kidney and activating blood circulation. It consists of six herbs: *Astragali radix*, *Angelicae sinensis radix*, *Ligustici Chuanxiong Rhizoma*, *Cuscutae semen*, *Taxilli Herba*, and *Dipsaci Radix*, and the main active constituents of BSHXR are ferulic acid, calycosin-7-glucopyranoside, hyperoside, quercitrin, and asperosaponin VI. In clinical practice, BSHXR is traditionally used to treat failed pregnancy and its complications. However, little is known about the underlying mechanism of BSHXR for the treatment of implantation loss during early pregnancy. In the current study, controlled ovarian hyperstimulation was induced in mice as our implantation loss model, and we evaluated the effects of BSHXR on implantation, decidualization, decidual angiogenesis, and reproductive outcome. We showed that BSHXR could regulate the supraphysiological levels of serum estrogen and progesterone observed in these mice, and also act on estrogen and progesterone receptors in the stroma and epithelium. BSHXR also enhanced FGF2 expression in the vascular sinus folding area of the decidua, thus potentially reducing implantation loss during early pregnancy and contributing to placentation and survival of the fetuses. Taken together, our findings provide scientific evidence for the application of BSHXR in the clinic as a treatment for implantation loss during early pregnancy, and warrant further investigation of BSHXR as an effective treatment for failed pregnancy and its complications.

## Introduction

Infertility is faced by approximately 15% of couples worldwide and is becoming a growing social and economic concern for many countries (Wang and Dey, 2006). In humans, the chances of natural conception during one menstrual cycle is around 30%, and only 50–60% of all conceptions progress beyond 20 weeks of gestation. Although the development of *in vitro* fertilization andembryo transfer (IVF-ET) technology has contributed significantly to the treatment of infertility, the pregnancy rate and delivery rate remain relatively low in IVF-ET cycles (European IVF-Monitoring Consortium [EIM] et al., 2016).

With a history of 2,000 years in East Asia, Chinese medicine has become popular for the prevention and treatment of various diseases, not only in China but also globally. According to a systematic review of published literature regarding Chinese medicines, 339,792 publications focusing on the study of Chinese medicines were identified until 15 April 2013, however, only 12,912 (3.8%) papers studied pregnancy or pregnancy related applications (Li et al., 2014). Among the literature about traditional Chinese medicines and pregnancy, it is discovered that Chinese medicines could affect various aspects of pregnancy, such as hormone regulation, endometrial receptivity, immune responses, infertility stress, and reproductive outcome of IVF-ET (Huang and Chen, 2008).

Based on the theories of traditional Chinese medicine, failed pregnancy can be attributed to kidney deficiencies and the stagnation of blood, which will lead to spontaneous abortion in patients. Thus, nourishing the kidneys and stimulating blood circulation are effective measures for the treatment of pregnancy loss. Many clinical observations have demonstrated that Bushen (nourishing kidney) and Huoxue (activating blood circulation) treatment could increase endometrial receptivity and pregnancy rate (Ma et al., 2015), improve follicular development and thickness of the endometrium (Jiang et al., 2006), and alleviate luteal function deficiencies in patients (Yang, 2004). In combination with IVF-ET, nourishing the kidney and activating blood circulation could increase both fertilization rate and pregnancy rate (Li, 2008). This was also observed in mice (Yang et al., 2001).

Bushen Huoxue recipe (BSHXR) is a widely used herbal prescription for nourishing the kidney and stimulating blood circulation. It has traditionally been applied to the treatment of recurrent spontaneous abortion and significant efficacy for preventing pregnancy loss is observed in clinic. According to the previous studies in rats, Bushen Huoxue decoction could promote LIF expression during the oestrous cycle which is important for implantation (Gong et al., 2013), and markedly increase the number of live births (Gong et al., 2015). However, more basic research is needed for exploring the mechanisms underlying the pro-fertility effect of BSHXR.

In light of the general traditional Chinese medicine theories, we hypothesized that nourishing the kidney may refer to the regulatory role of BSHXR on ovarian hormones and their receptors as it is believed that kidney in Chinese medicine theory is in control of the production and regulation of reproductive hormones, and activating blood circulation may indicate that BSHXR could modulate the development and maturation of the endometrial vascular network during pregnancy. Therefore, in the present study, we firstly established an early pregnancy loss mouse model by controlled ovarian hyperstimulation (COH), and investigated the expression of hormone receptors: ERα and PR, and three most important angiogenic factors: VEGFA, ANGPT2, and FGF2 at different time points during early pregnancy. We showed that BSHXR could reduce post-implantation fetal loss through regulation of estrogen–progesterone signals and decidual angiogenesis during early pregnancy, thereby offering a treatment for early pregnancy loss.

## Materials and Methods

### BSHXR Preparation and HPLC Analysis

Bushen Huoxue recipe was provided by HuaRun SanJiu Medical and Pharmaceutical Co., Ltd. (Shenzhen, China). The six herbal components of BSHXR were presented (**Table 1**), which were mixed at a weight ratio of 7:6:6:7:6:6. The voucher specimens were deposited at the Research and Development Center of China Resources SanJiu Medical and Pharmaceutical Co., Ltd. (Shenzhen, China), and authenticated by Anhui University of Chinese Medicine (Anhui, China). The six herbs were crushed into crude grains and a solution was achieved by refluxing with boiling water. The solution was subsided by ethanol and granules were obtained through concentration and vacuum-drying. One gram dry BSHXR granule is equivalent to 10 g *Astragali radix*, 3.33 g *Angelicae sinensis radix*, 6 g *Ligustici Chuanxiong Rhizoma*, 20 g *Cuscutae semen*, 15 g *Taxilli Herba*, and 6.67 g *Dipsaci Radix* raw medical herbs. In the present study, granules were dissolved in boiling water and stirred evenly before use. Herbal granules were stored under dry condition in 4°C.

**Table 1:** The compositions of BSHXR.

Chinese name	Latin name	Family	Voucher number	Weight (g)	Part used
Huang qi	*Astragali radix*	*Astragalus aaronii* (Eig) Zohary	J4013-6115	14	Root
Dang gui	*Angelicae sinensis radix*	*Angelica sinensis* (Oliv.) Diels	J4012-6111	12	Root
Chuan xiong	*Ligustici Chuanxiong Rhizoma*	*Ligusticum acuminatum* Franch.	J4013-6128	12	Root
Tu si zi	*Cuscutae semen*	*Cuscuta chinensis* Lam.	J4013-6119	14	Seed
Sang ji sheng	*Taxilli Herba*	*Loranthus acaciae* Zucc.	J4013-6131	12	Stem and branch
Xu duan	*Dipsaci Radix*	*Dipsacus acaulis* (A.Rich.) Napper	J4013-6132	12	Root

To ensure the quality and stability of BSHXR, we used high performance liquid chromatography (HPLC) to identify the active compounds in BSHXR, and three batches were examined. The batch numbers were as follows: *Astragali radix* (1503001W, 1503001S, and 1604001W), *Angelicae sinensis radix* (1412005W, 1412002S, and 1604007S), *Ligustici Chuanxiong Rhizoma* (1607002W, 1507002S, and 1608001S), *Cuscutae semen* (1505001S, 1507001S, and 1607001S), *Taxilli Herba* (1508001W, 1404001W, and 1607001W), and *Dipsaci Radix* (1608001W, 1508001S, and 1607001W). The substances used for the preparation of reference solution were purchased from China National Institutes for Food and Drug Control (Beijing, China). HPLC was performed on Waters 1525-2489 HPLC system (Milford, CT, United States), and Waters SunFire C_18_ column (250 mm × 4.6 mm, 5 μm) was used for analysis. The mobile phase consisted of acetonitrile (A) and 0.04% phosphoric acid (B) in gradient elution mode as following: 0–65 min, 10%–28% A, 90%–72% B; 65–80 min, 28%–45% A, 72%–59% B. The flow velocity was 1.0 ml/min and the temperature of the column was maintained at 30°C. The injection volume was 20 μl.

### Animal Treatments and Sample Collection

**FIGURE 1:**
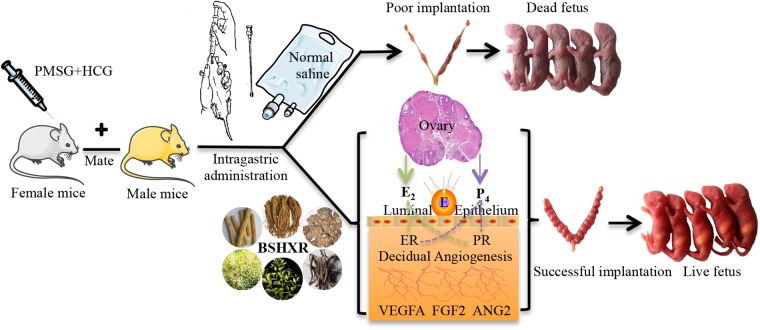
The graphic illustration of experiment. E, embryo; ER, estrogen receptor; PR, progesterone receptor.

The overall graphic scheme is summarized (**Figure 1**). Mice of the Kun-ming breed (8-week-old female mice and 10-week-old male mice) were purchased from the Laboratory Animal Center of Tongji Medical College, Huazhong University of Science and Technology. Approval of all experimental procedures was obtained from the Tongji Medical College Ethics Committee (Huazhong University of Science and Technology).

The mice were caged in a specific pathogen-free environment with 12 h light:12 h darkness rhythm and fed *ad libitum*. Female mice were randomly divided into four groups as follows: control group, model group, BSHXR1 (normal dose 11.4 g/kg) group, and BSHXR2 (low dose 5.7 g/kg) group. After acclimation for 1 week without any experimental intervention, female mice were inspected daily for vaginal smears. In the model group, the BSHXR1 group and the BSHXR2 group, COH was induced in female mice at diestrus with an intraperitoneal injection of 7.5 IU pregnant mare serum gonadotrophin (PMSG; Hangzhou Animal Medicine Factory, China) followed by 7.5 IU of human chorionic gonadotropin (hCG; Lizhu Pharmaceutical Factory, China) 48 h later. After superovulation, female mice were caged with male mice overnight for mating at a ratio of 1:1. In the control group, female mice were injected with normal saline at the appropriate times and mated with male mice during estrus at a ratio of 1:1. For all groups, a vaginal plug on the next morning was taken as evidence of successful mating and that day was considered to be day 1 of gestation (D1).

From D1, the vaginal plug-positive female mice of the control and model groups were given 0.3 ml normal saline daily by oral gavage. As for BSHXR1 and BSHXR2 groups, 11.4 g/kg (normal dose) and 5.7 g/kg (low dose) BSHXR was administered daily by oral gavage. The normal dose of BSHXR is 76 g/day for an adult weighing 60 kg in the clinic, thus we calculated the normal dose of BSHXR for mice according to the conventional method (Wei et al., 2010) The BSHXR granules were dissolved in boiling water and stirred evenly to room temperature before use.

On D5, D6, and D8, some of the vaginal plug-positive female mice were sacrificed, and pregnant mice were identified by the morphology of the uterus. The numbers of implantation sites on D5, D6, and D8 in the four treatment groups were recorded. Blood samples were collected and centrifuged at 3000 ×*g* for 15 min at 4°C. After centrifugation, serum was separated and stored at -80°C for measurement of hormones. Ovaries and uterus were removed for morphological analysis and quickly frozen for RNA and protein extraction.

To further study the effects of BSHXR on pregnancy outcome, some vaginal plug-positive female mice from each group was kept until spontaneous delivery and the overall condition of the fetuses were observed. We recorded the number of live/dead fetuses per pregnancy and the lengths of gestation period. Ultimately, the pregnancy rate and the rate of post-implantation pregnancy loss were calculated as previously described (Bindali and Kaliwal, 2002; Rudge et al., 2007).

### Morphological Assessment of the Ovary and Uterus

For morphological analysis, the ovaries and uterus were collected and fixed in 4% paraformaldehyde (PFA) overnight, dehydrated and then embedded in paraffin. Hematoxylin and eosin staining (HE staining) was performed on ovary and uterus sections. Images were obtained from an Olympus DP73 upright microscope (Olympus, Tokyo, Japan) with the CellSens Standard acquisition software.

### Analysis of Serum Hormone Levels

The serum levels of estrogen and progesterone were measured by radioimmunoassay (RIA) (Beijing North Institute of Biological Technology, Beijing, China) according to the manufacturer’s instructions.

### Quantitative Real-Time PCR

After removing the embryos, total RNA was extracted from the homogenized decidual tissue using RNAiso Plus (TaKaRa) according to the manufacturer’s instructions. The concentration and purity of RNA was measured by a nucleic acid/protein analyzer (Thermo, Rockford, IL, United States). cDNA synthesis was conducted with a PrimeScript RT reagent kit (TaKaRa) in a reaction volume of 20 μl. Quantitative Real-Time PCR (qRT-PCR) analysis was carried out using SYBR Green qPCR kit (TaKaRa) and Applied Biosystems StepOne Real-Time PCR System (Applied Biosystems) according to the manufacturer’s instructions. The PCR conditions were as follows: initial denaturation for 30 s at 95°C, 40 cycles of 5 s at 95°C, annealing and elongation for 30 s at 60°C. Each sample was run in triplicate (*n* = 5–6 per group) and β-actin was used as the normalization reference. The primer sequences originated from NCBI GenBank database and had been previously verified. The specific primer sequences are listed in **Table 2**. Fold-change of gene expression was calculated using 2^-ΔΔC_T_^ method (Livak and Schmittgen, 2001; Schmittgen and Livak, 2008).

**Table 2:** Primer sequences.

Gene name	Forward (5′–3′)	Reverse (5′–3′)
*Esr1*	CAAGGTAAATGTGTGGAAGGCA	ATGGGAAAGAATGAGAAGGAGC
*Pgr*	GTCACTATGGCGTGCTTACCTG	CAACACCGTCAAGGGTTCTCAT
*Vegfa*	AGGAGTACCCCGACGAGATAGA	CACATCTGCTGTGCTGTAGGAA
*Angpt2*	GTCCATGAAGGAGCAGAAGG	GCCTTGATCTCCTCTGTGGA
*Fgf2*	GCTGCTGGCTTCTAAGTGTGTT	TCGTTTCAGTGCCACATACCA
*β-actin*	GTGACGTTGACATCCGTAAAGA	GTAACAGTCCGCCTAGAAGCAC

### Western Blot Analysis

Decidual tissues with the embryo removed were lysed in RIPA lysis buffer with protease inhibitors (Goodbio Technology Co., Wuhan, China). Then, lysates were centrifuged at 12,000 ×*g* for 10 min and the protein concentration of the supernatants were measured by BCA assay kit (Goodbio Technology Co., Wuhan, China). Protein samples were separated on 10% sodium dodecyl sulfate-polyacrylamide gel electrophoresis (SDS-PAGE) and transferred onto PVDF membranes. After transferring, the PVDF membranes were blocked with 5% non-fat dry milk in 0.5% TBS-Tween for 1 h at room temperature. Anti-ERα (sc-542; 1:1000; Santa Cruz Biotechnology), anti-PR (ab133526; 1:1000; Abcam), anti-VEGFA (AF-493-NA; 1:1000; R&D Systems), anti-ANGPT2 (ab155106; 1:1000; Abcam), anti-FGF2 (sc-79; 1:100; Santa Cruz Biotechnology), and anti-β-actin (GB13001-1; 1:1000; Goodbio Technology Co, Wuhan, China) antibodies were used for incubation overnight at 4°C. After the incubation of primary antibodies overnight, the membranes were washed with TBST three times for 10 min each, and incubated with fluorescent secondary antibody (P/N 926-32211, 1:5000; Odyssey) for 1 h at room temperature. Bands were visualized with Odyssey Infrared Image System (LI-COR, Lincoln, NE, United States), and ImageJ software (National Institutes of Health, United States) was used to quantify the relative protein expression. All assays were performed three times independently and the raw data of three independent experiments are shown in Supplementary Figure S1.

### Immunohistochemistry (IHC)

Paraffin sections of the uterus were de-paraffinized in xylene and rehydrated in declining concentrations of ethanol. Next, antigen retrieval was performed with citric acid buffer (pH = 6.0) at 95°C for 20 min. Then, sections were incubated with 3% H_2_O_2_ for 25 min at room temperature to inhibit endogenous peroxidase activity, and then blocked in 10% rabbit serum for 30 min at room temperature. Anti-CD31 (ab28364; 1:50; Abcam), Anti-ERα (sc-542; 1:50; Santa Cruz Biotechnology), anti-PR (sc-538; 1:50; Santa Cruz Biotechnology), anti-VEGFA (AF-493-NA; 1:100; R&D Systems), anti-ANGPT2 (sc-20718; 1:50; Santa Cruz Biotechnology), and anti-FGF2 (sc-79; 1:100; Santa Cruz Biotechnology) antibodies were used as primary antibodies for incubation overnight at 4°C, followed by washing 5 min three times in PBS. Tissue sections were then incubated with the corresponding secondary antibody (GB23204; 1:200; Goodbio Technology Co.) for 50 min at room temperature. After developing with 3,3′-diaminobenzidinetetrahydrochloride (DAB), cell nuclei were counterstained using Harris hematoxylin for 3 min. Finally, the sections underwent dehydration and were sealed with neutral balsam. Immunohistochemical pictures were taken using an Olympus DP73 upright microscope (Olympus, Tokyo, Japan) with the CellSens Standard acquisition software. Cell nuclei were stained blue and yellowish-brown staining was considered positive.

### Statistical Analysis

Data with a normal distribution were represented as mean ± SD, and data with a skewed distribution were represented as medians (first quartile, third quartile). All data were analyzed by SPSS 20.0 software (SPSS Inc., Chicago, IL, United States). One-way analysis of variance (ANOVA) was used to compare differences amongst the four groups, more specifically, a LSD or Dunnett’s T3 test was used depending on equal variances of data. Furthermore, Chi-square test was applied for comparison of rate. A *p*-value < 0.05 was considered statistically significant.

## Results

### HPLC Profile of BSHXR

**FIGURE 2:**
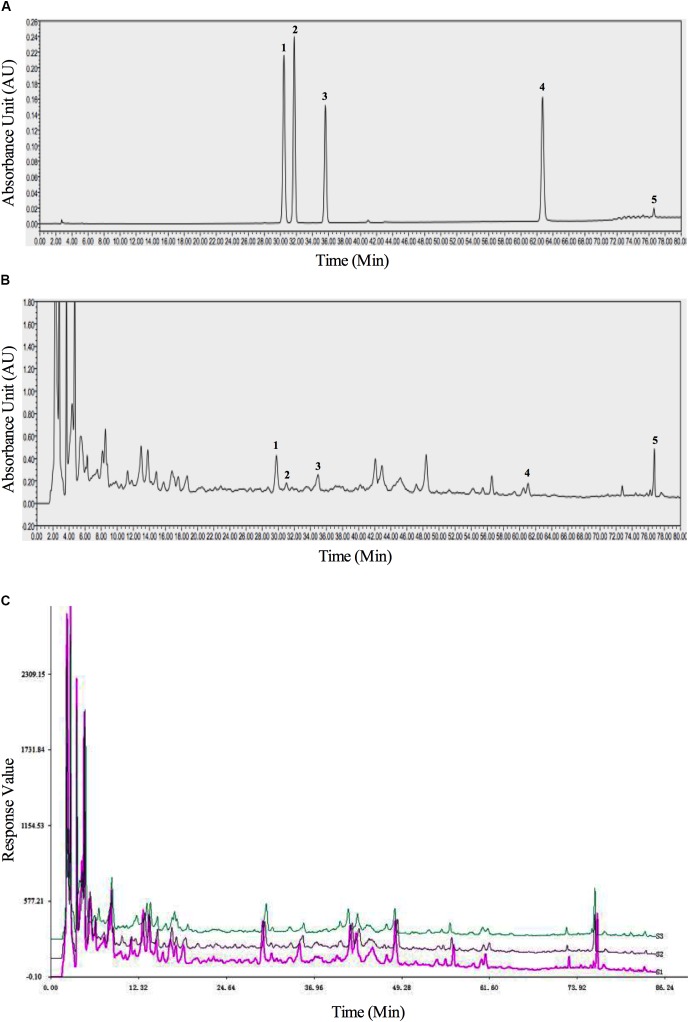
The HPLC fingerprints of reference standards **(A)**, BSHXR **(B)**, and BSHXR from three batches **(C)**. Peak number and identity: 1, ferulic acid; 2, calycosin-7-Glucopyranoside; 3, hyperoside; 4, quercitrin; 5, asperosaponin VI.

BSHXR consists of six herbs (composition summarized in **Table 1**). The qualitative determination of BSHXR was performed by HPLC. Five peaks were identified by comparing the retention times with the reference standard, and the main constituents of BSHXR were as follows: ferulic acid; calycosin-7-glucopyranoside; hyperoside; quercitrin; asperosaponin VI. Their retention times were 30.50 min, 31.75 min, 35.75 min, 62.75 min, and 76.50 min, respectively (**Figures 2A,B**). Three batches of BSHXR were tested by HPLC (**Figure 2C**).

### Effects of BSHXR on Implantation and Reproductive Outcomes

Implantation and reproductive outcomes were observed in our study (**Table 3**). There were no significant differences in pregnancy rate, number of implantation sites on D5, D6, D8 and number of live fetus between the four groups. However, the number of live fetuses was highly variable in the model group, and post-implantation pregnancy loss was remarkably elevated in the model group compared to control (*P* < 0.05). BSHXR1 treatment reduced post-implantation pregnancy loss significantly (*P* < 0.05) while BSHXR2 treatment also reduced pregnancy loss, but not significantly. Moreover, compared with the increased number of dead fetuses in the model group, no dead fetuses were observed in the other three groups (*P* < 0.05). The gestational period of the model group was significantly longer than the control group (*P* < 0.001), and both BSHXR groups could shorten the gestation period to the normal length as that of the control group (*P* < 0.001).

**Table 3:** Effects of BSHXR on implantation and pregnancy outcome in mice.

Groups	Control	Model	BSHXR1 (11.4 g/kg)	BSHXR2 (5.7 g/kg)
Pregnancy rate (%)	71.43 (30/42) × 100	55.56 (30/54) × 100	65.22 (30/46) × 100	55.36 (31/56) × 100
No. of implantation on D5 (*N* = 10)	13.50 ± 1.58	18.90 ± 2.73	14.90 ± 3.73	15.30 ± 2.00
No. of implantation on D6 (*N* = 10)	13.10 ± 1.79	17.50 ± 4.25	13.80 ± 2.62	14.30 ± 3.47
No. of implantation on D8 (*N* = 10)	12.00 ± 2.87	11.60 ± 3.47	13.50 ± 2.01	13.00 ± 3.74
No. of live fetuses (*N* = 6)	11.67 ± 1.21	7.50 ± 6.72	12.00 ± 2.10	11.17 ± 3.55
No. of dead fetuses (*N* = 6)	0	4 (0,8.5)^∗^	0	0
Gestation period (days) (*N* = 6)	20.00 ± 0.63	22.00 ± 0.89^∗∗∗^	20.00 ± 0.00^###^	20.00 ± 1.10^###^
Post-implantation Loss (%)	13.56 (13.50–11.67/13.50) × 100	60.32^∗^ (18.90–7.50/18.90) × 100	19.46^#^ (14.90–12/14.90) × 100	26.99 (15.30–11.17/15.30) × 100

*Data with a normal distribution represent as mean ± SD, and data with a skewed distribution represent as medians (first quartile, third quartile). ^∗^P < 0.05 and ^∗∗∗^P < 0.001 when compared to control group. ^#^P < 0.05 and ^###^P < 0.001 when compared to model group.*

**FIGURE 3:**
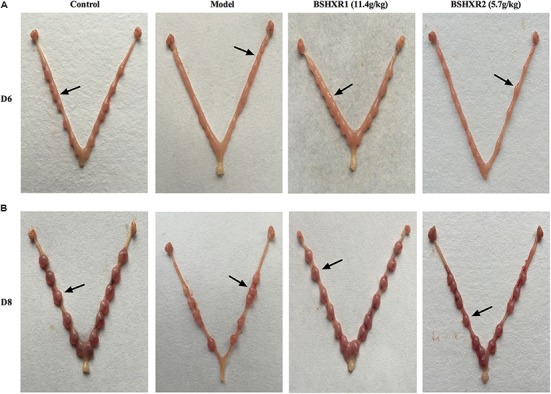
Visible external morphology of uterus showing implantations on D6 **(A)** and on D8 **(B)**. Arrows indicate implantation sites.

To further confirm the effects of BSHXR on implantation, we also compared the morphology of the pregnant uterus on D6 and D8 when the implantation sites were visible for comparison (**Figures 3A,B**). Compared with the well-nourished implantation sites in the control group, it was evident that the implantation sites in the model group were relatively smaller and poorly developed. Both doses of BSHXR could improve the development of implantation sites, but the effects of BSHXR1 were better than that of BSHXR2.

### Effects of BSHXR on Ovary Morphology and Sex Hormones

**FIGURE 4:**
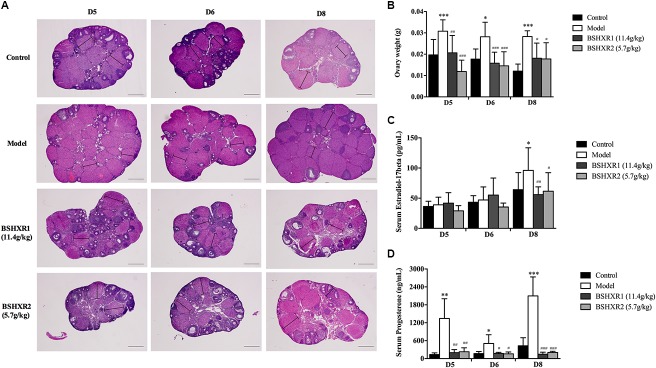
Effect of BSHXR on the morphology of ovary and serum hormones. **(A)** Morphology analysis of ovary section (HE, ×40), lines indicate maximum diameters of corpus luteum. Bars = 500 μm. **(B)** Measurement of bilateral ovary weight on D5, D6, and D8, *N* = 9–10. **(C)** Serum estradiol-17β levels on D5, D6, and D8, *N* = 8–10. **(D)** Serum progesterone levels on D5, D6, and D8, *N* = 8–10. Data represent as mean ± SD. ^∗^*P* < 0.05, ^∗∗^*P* < 0.01, and ^∗∗∗^*P* < 0.001 when compared to control group. ^#^*P* < 0.05, ^##^*P* < 0.01, and ^###^*P* < 0.001 when compared to model group.

Since the steroid hormones produced by the ovary are vital for implantation and early pregnancy, we analyzed the morphology and weight of the ovary, as well as the serum levels of estrogen and progesterone. In the HE section of the ovary, it was found that more corpora lutea were presented in the model group, and the volume and weight of the ovary was increased compared to the control group (*P* < 0.001 or *P* < 0.05) (**Figures 4A,B**), reflecting the hyperstimulated status of ovary due to COH. In contrast, BSHXR1 and BSHXR2 groups had less corpora lutea and decreased volume and weight of the ovary compared with those of the model group (*P* < 0.001 or *P* < 0.01 or *P* < 0.05). Furthermore, the serum level of estrogen was elevated in the model group on D8 when compared to the control group (*P* < 0.05) (**Figure 4C**), and both BSHXR1 and BSHXR2 treatments reduced the excessive increase in estrogen significantly (*P* < 0.01 or *P* < 0.05). Moreover, compared with the serum levels of progesterone in the control group, the model group exhibited a marked increase in progesterone at D5, D6, and D8 (*P* < 0.001 or *P* < 0.01 or *P* < 0.05) (**Figure 4D**), while BSHXR1 and BSHXR2 could reduce the secretion of progesterone to the normal serum levels (*P* < 0.001 or *P* < 0.01 or *P* < 0.05).

### Effects of BSHXR on Decidualization

**FIGURE 5:**
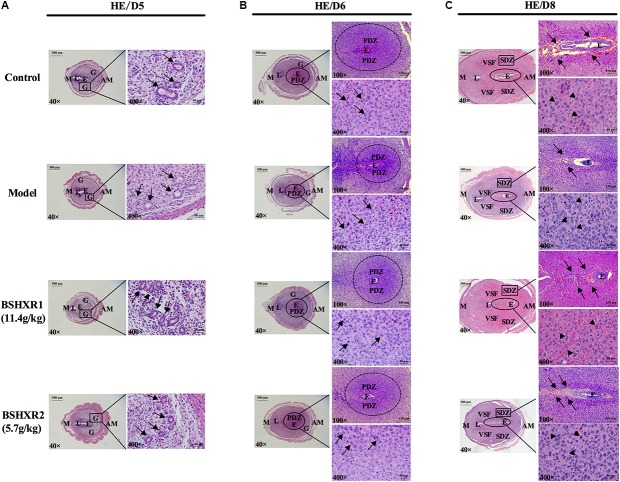
Morphology analysis of uterine section on D5, D6, and D8. The right pictures are the higher magnification images of the black boxes or ellipses on the left. **(A)** The development of glands on D5. Arrows indicate glands. **(B)** Formation of PDZ on D6. Arrows indicate decidual cells. **(C)** Formation of SDZ and surrounding blood vessel of embryo on D8. Arrows indicate blood vessels and arrowheads indicate decidual cells. E, embryo; L, lumen; G, gland; PDZ, primary decidual zone; SDZ, secondary decidual zone; M, mesometrial; AM, anti-mesometrial; VSF, vascular sinus folding. Original magnifications and scale bar were shown in images.

In order to observe the effects of BSHXR on implantation and subsequent decidualization, we performed morphological assessment of uterine tissue sections on D5, D6, and D8. On D5, the glandular cells were cuboidal in the model group compared to columnar in the control and BSHXR groups (**Figure 5A**). On D6 and D8, the cytoplasm of decidual cells in the primary decidual zone (PDZ) and second decidual zone was reduced in the model group compared to the control group, while BSHXR1 and BSHXR2 could increase the content of cytoplasm in decidual cells (**Figures 5B,C**). Apart from the alteration of cytoplasmic content in decidual cells on D6 and D8, it was also discovered that the blood vessels around the embryo were remarkably diminished in the model group on D8 compared with the control group. Both BSHXR1 and BSHXR2 could increase the blood vessels around the embryo, though the effect of BSHXR1 was better than that of BSHXR2 (**Figure 5C**).

### Effects of BSHXR on ERα and PR Expression

**FIGURE 6:**
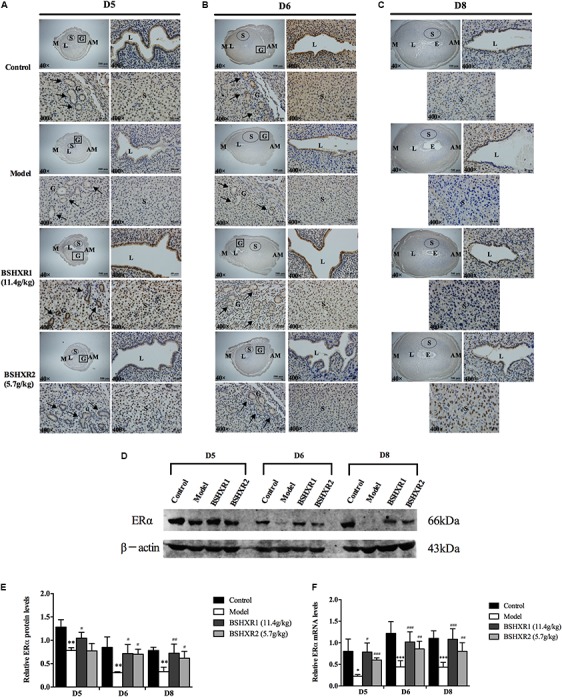
ERα expression in the uterus on D5, D6 and D8. **(A)** Immunohistochemistry of ERα in paraffin sections of the uterus on D5 **(A)**, D6 **(B)**, and D8 **(C)**. For each group (control, model, BSHXR1, and BSHXR2), a low magnification image of the uterus is depicted, as well as higher magnification images of the lumen (L), gland (G), and stroma (S). E, embryo; M, mesometrial; AM, anti-mesometrial; L, lumen; G, gland; S, stroma. Arrows indicate glands. Original magnification and scale bar were shown in images. **(D)** Protein levels of ERα in pregnant uterus (remove embryo) by western blot analysis (*n* = 3). **(E)** Quantification of ERα protein expression. **(F)** mRNA levels of ERα in pregnant uterus (remove embryo) by qRT-PCR (*n* = 5). Data represent mean ± SD. β-actin was used as the reference. ^∗^*P* < 0.05, ^∗∗^*P* < 0.01, and ^∗∗∗^*P* < 0.001 when compared to control group. ^#^*P* < 0.05, ^##^*P* < 0.01, and ^###^*P* < 0.001 when compared to model group.

**FIGURE 7:**
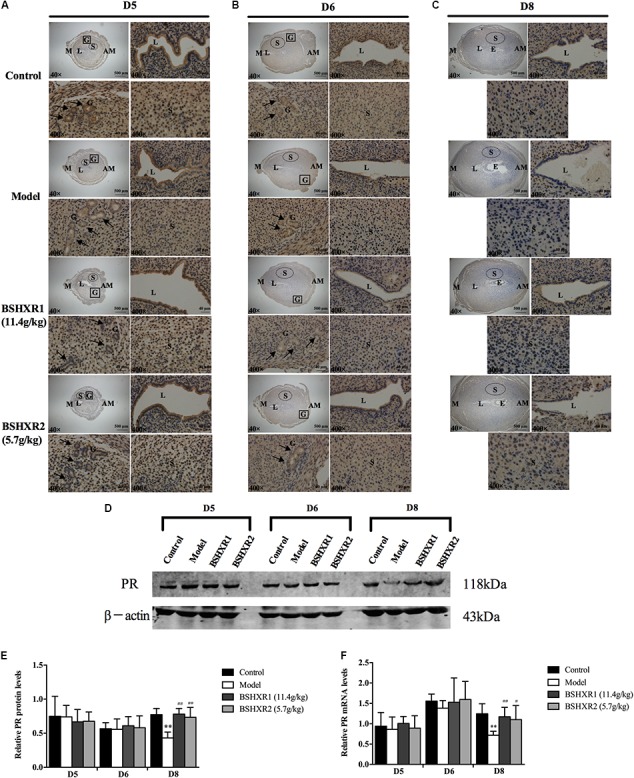
PR expression in the uterus on D5, D6 and D8. **(A)** Immunohistochemistry of PR in paraffin sections of the uterus on D5 **(A)**, D6 **(B)**, and D8 **(C)**. For each group (control, model, BSHXR1, and BSHXR2), a low magnification image of the uterus is depicted, as well as higher magnification images of the lumen (L), gland (G) and stroma (S). E, embryo; M, mesometrial; AM, anti-mesometrial; L, lumen; G, gland; S, stroma. Arrows indicate glands. Original magnification and scale bar were shown in images. **(D)** Protein levels of PR in pregnant uterus (remove embryo) by western blot analysis (*n* = 3). **(E)** Quantification of PR protein expression. **(F)** mRNA levels of PR in pregnant uterus (remove embryo) by qRT-PCR (*n* = 5). Data represent mean ± SD. β-actin was used as the reference. ^∗∗^*P* < 0.01 when compared to control group. ^#^*P* < 0.05 and ^##^*P* < 0.01 when compared to model group.

To further explore the underlying mechanism of BSHXR’s pro-implantation effect, we measured the mRNA and protein expression of ERα and PR in the decidua and their localization in the pregnant uterus (**Figures 6**, **7**). It was noticed that on D5 and D6, ERα was mainly expressed in stroma, luminal and glandular epithelium (**Figures 6A,B**). In the model group, the expression of ERα was reduced in the stroma, luminal and glandular epithelium on D5 and D6 compared to the control group, and this was prevented by BSHXR1 and BSHXR2 treatment. Again, the effect of BSHXR1 was better than that of BSHXR2.

On D8, ERα was mainly expressed in the stroma and luminal epithelium (**Figure 6C**). In the model group, the expression of ERα was reduced in the stroma on D8 compared to the control group, and this could be prevented by BSHXR1 and BSHXR2. In addition to the reduction of ERα protein expression, mRNA expression was also reduced in the model group (*P* < 0.01) when compared to the control group on D5, D6, and D8, and BSHXR1 could significantly increase ERα mRNA and protein expression on D5, D6, and D8 (*P* < 0.001 or *P* < 0.01 or *P* < 0.05). Although the mRNA expression of ERα was increased in the BSHXR2 group on D5, the protein level was not different compared to the model group. On D6 and D8, BSHXR2 significantly increased the mRNA and protein levels of ERα (*P* < 0.001 or *P* < 0.01 or *P* < 0.05) (**Figures 6D–F**).

Additionally, it was discovered that PR was mainly expressed in the stroma, and the luminal and glandular epithelium on D5 and D6 (**Figures 7A,B**). On D5, a slight reduction in the luminal epithelial PR expression in the model group was observed, but this was improved in the BSHXR groups (**Figure 7A**). On D6, there was no notable difference in PR expression in the stroma or the epithelium between the four groups (**Figure 7B**). On D8, PR expression in the stroma was reduced in the model group compared with the control group, and both BSHXR1 and BSHXR2 could increase stromal expression (**Figure 7C**). However, quantification of protein and transcript levels of PR showed that there was no difference in PR protein or mRNA expression between the four groups on D5 and D6 (**Figures 7D–F**), while on D8, PR mRNA and protein levels were reduced in the model group (*P* < 0.01) compared with the control group. Both BSHXR1 and BSHXR2 could significantly raise the mRNA and protein levels of PR on D8 (*P* < 0.01 or *P* < 0.05).

### Effects of BSHXR on Decidual Angiogenesis

**FIGURE 8:**
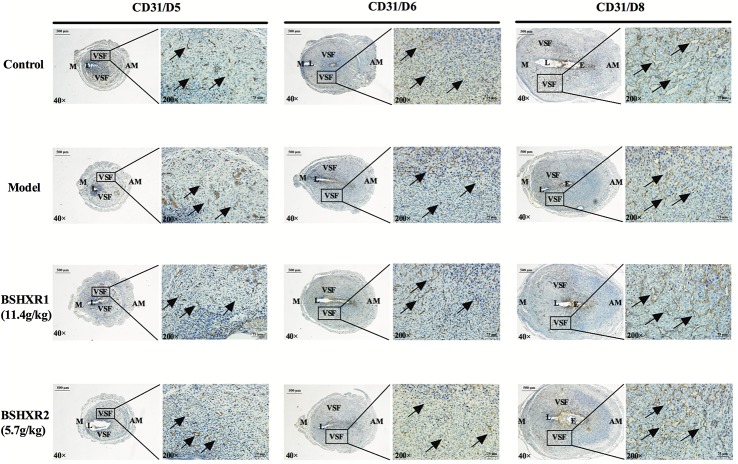
Decidual angiogenesis of the pregnant uterus on D5, D6, and D8. Immunohistochemistry staining with cluster of differentiation 31 (CD31). The pictures on the right are the higher magnification images of VSF. Arrow represents the variable-sized blood vessels. E, embryo; L, lumen; M, mesometrial; AM, anti-mesometrial; VSF, vascular sinus folding. Original magnification and scale bar were shown in the pictures.

Since we found defective blood vessel formation around the embryo in the model group, we further investigated the effects of BSHXR on decidual angiogenesis in pregnant mice. As shown by HE staining, on D8, blood vessels were significantly reduced in the model group compared to the control group, and abnormalities in vascular density and morphology of the vascular sinus folding (VSF) were observed (**Figure 8**). In the decidua of control animals, the sprouting of blood vessels became active on D6, and the enlargement and elongation of the VSF was boosted on D8. Compared with the control group, a decrease in vascular density and defective enlargement and elongation of the VSF from D6 to D8 were observed in the model group, while BSHXR could increase vascular density and promote the development of blood vessels, though the effect of BSHXR1 was better than BSHXR2.

### Effects of BSHXR on the Expression of VEGFA, ANG2, and FGF2

**FIGURE 9:**
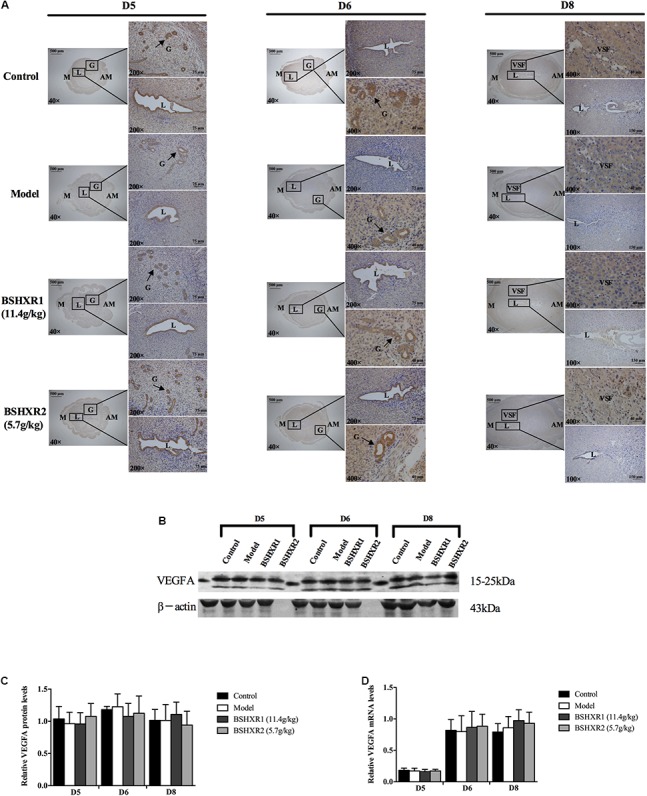
VEGFA expression in the uterus on D5, D6, and D8. **(A)** Immunohistochemistry of VEGFA in paraffin sections of the uterus. The right pictures are the higher magnification images of the black boxes on the left. Arrow indicates the positive staining. L, lumen; G, gland; M, mesometrial; AM, anti-mesometrial; VSF, vascular sinus folding. Original magnification and scale bar were shown in the pictures. **(B)** Protein levels of VEGFA in pregnant uterus (remove embryo) by western blot analysis (*n* = 3). **(C)** Quantification of VEGFA protein expression. **(D)** mRNA levels of VEGFA in pregnant uterus (remove embryo) by qRT-PCR (*n* = 5). Data represent mean ± SD. β-actin was used as the reference.

**FIGURE 10:**
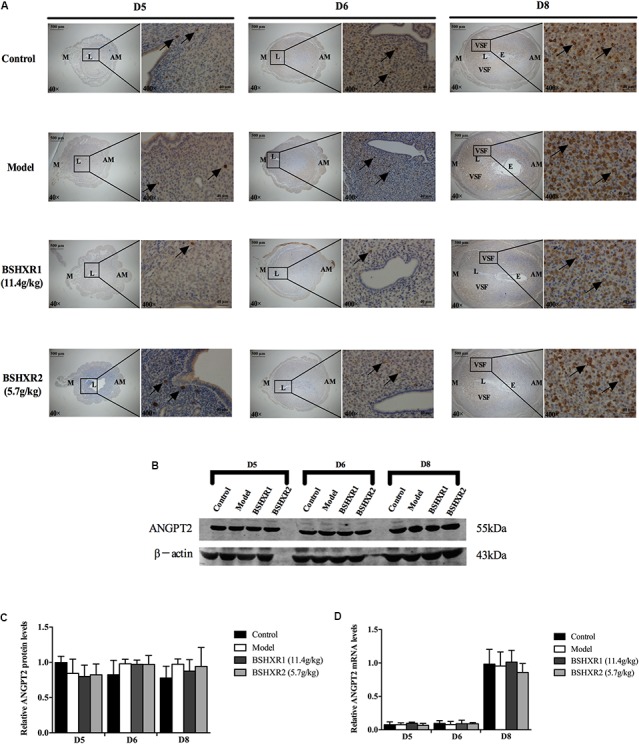
ANGPT2 expression in the uterus on D5, D6, and D8. **(A)** Immunohistochemistry of ANGPT2 in paraffin sections of the uterus. The right pictures are the higher magnification images of the black boxes on the left. Arrow indicates the positive staining. L, lumen; E, embryo; M, mesometrial; AM, anti-mesometrial; VSF, vascular sinus folding. Original magnification and scale bar were shown in the pictures. **(B)** Protein levels of ANGPT2 in pregnant uterus (remove embryo) by western blot analysis (*n* = 3). **(C)** Quantification of ANGPT2 protein expression. **(D)** mRNA levels of ANGPT2 in pregnant uterus (remove embryo) by qRT-PCR (*n* = 5). Data represent mean ± SD. β-actin was used as the reference.

**FIGURE 11:**
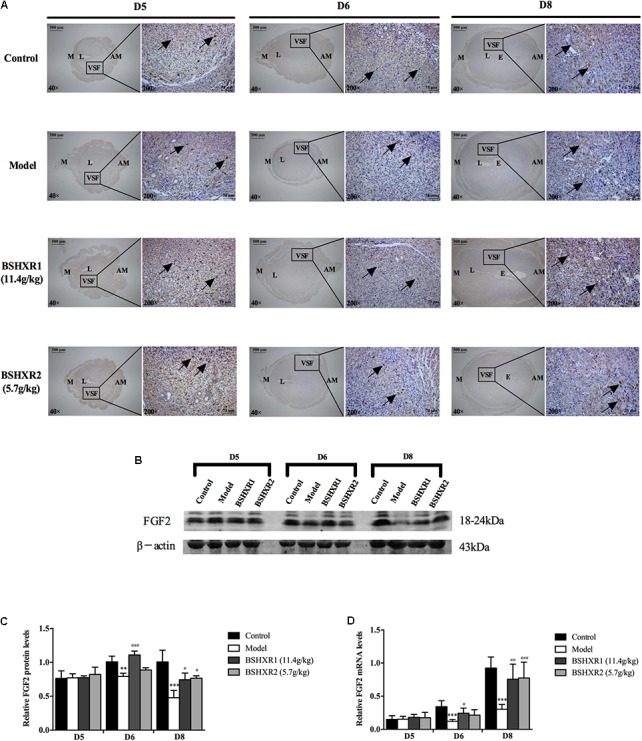
FGF2 expression in the uterus on D5, D6, and D8. **(A)** Immunohistochemistry of FGF2 in paraffin sections of the uterus. The right pictures are the higher magnification images of the black boxes on the left. Arrow indicates the positive staining. L, lumen; E, embryo; M, mesometrial; AM, anti-mesometrial; VSF, vascular sinus folding. Original magnification and scale bar were shown in the pictures. **(B)** Protein levels of FGF2 in pregnant uterus (remove embryo) by western blot analysis (*n* = 3). **(C)** Quantification of FGF2 protein expression. **(D)** mRNA levels of FGF2 in pregnant uterus (remove embryo) by qRT-PCR (*n* = 5). Data represent mean ± SD. β-actin was used as the reference. ^∗∗^*P* < 0.01 and ^∗∗∗^*P* < 0.001 when compared to control group. ^#^*P* < 0.05, ^##^*P* < 0.01, and ^###^*P* < 0.001 when compared to model group.

Representative immunohistochemical staining of VEGFA, ANG2, and FGF2 in the pregnant uterus is presented in **Figures 9A**, **10A**, **11A**, respectively. VEGFA was mainly distributed in the luminal and glandular epithelium on D5 and D6, with some expression in the stroma; while by D8, VEGFA has almost disappeared from the luminal epithelium and is present only in the stroma. At the mRNA level, VEGFA expression was significantly increased on D6 compared to D5, coinciding with the sprouting of blood vessels that also became evident at D6. This supports a role of VEGFA in decidual angiogenesis. There was no notable difference in VEGFA mRNA and protein expression from D5 to D8 between the four groups (**Figures 9B–D**).

Furthermore, ANG2 was mainly expressed in the mesometrial region and its expression continually increased from D5 to D8, and levels were especially high in the VSF region on D8. Low levels of ANG2 mRNA was observed on D5 and D6, but it was highly induced on D8. As the enlargement and elongation of the VSF was dominant on D8, this suggests that ANG2 may play an important role in the maturation of decidual blood vessels. There was no notable difference in ANG2 mRNA and protein expression from D5 to D8 between the four groups (**Figures 10B–D**).

As for FGF2, its expression was mainly located at the VSF region and peaked on D8. At the mRNA level, FGF2 expression increased from D5 to D8 in the control group, and FGF2 mRNA and protein expression was decreased in the model group compared with the control group on D6 and D8. BSHXR1 significantly promoted FGF2 mRNA and protein expression on D6 and D8, however, BSHXR2 could only significantly elevate FGF2 mRNA and protein expression on D8 (**Figures 11B–D**).

## Discussion

Pregnancy is a very complex process, consisting of inseparable events including implantation, decidualization, placentation, and final parturition (Carson et al., 2000; Dey et al., 2004; Wang and Dey, 2006). In mice, embryo implantation occurs at midnight of day 4 (day 1 = day of vaginal plug), and after implantation, the embryo directly communicates with the endometrium and decidualization is initiated to protect the embryo (Zhang et al., 2013). Upon decidualization, stromal cells undergo polyploidization, with extensive proliferation and differentiation characterized by cell enlargement, expansion of organelles and cytoplasmic accumulation of glycogen and lipid droplets (Gellersen et al., 2007). On the afternoon of day 5, an avascular PDZ is formed and then stromal cells around the PDZ continue to proliferate and differentiate for the establishment of the secondary decidual zone (SDZ) by day 8 (Zhang et al., 2013). A well-developed decidua is vital for the survival of the developing embryo and the regulation of trophoblast invasion before the formation of the placenta (Welsh and Enders, 1987; Wang et al., 2004; Peng et al., 2008). Therefore, decidualization is considered to be crucial for pregnancy establishment and maintenance. Despite the rapid development of IVF-ET, the rate of early pregnancy loss after implantation remains high, around 25–40% (Wilcox et al., 1988). However, given the complexities of early pregnancy development, genetic abnormalities, hormone regulation, decidual angiogenesis, immunologic factors, and many other mechanisms are likely to be involved (Norwitz et al., 2001).

The clinical application of Chinese herbal medicine for pregnancy complications is becoming popular, not only in East Asia but also in Western countries. With the extensive application of Chinese medicine as an adjunct in assisted reproduction technology nowadays, it has been demonstrated that IVF with Chinese medicine can improve live birth rates in donor and non-donor cycles (Hullender Rubin et al., 2015). Also one study reported that when compared with western fertility drug therapy or IVF, the application of Chinese herbal medicine could increase pregnancy rate twofold within a 4-month period (Ried and Stuart, 2011). BSHXR is one such effective prescription for the treatment of pregnancy complications. BSHXR is established upon the treatment principles of nourishing the kidneys and activating blood circulation according to traditional Chinese medicine theories. In our present study, we established a COH mice model for early pregnancy loss by administering PMSG and HCG. In accordance with previous descriptions, defects in implantation and impaired decidualization were discovered in COH mice (Deng et al., 2013; Ezoe et al., 2014), and we found that BSHXR could decrease post-implantation fetal loss in COH mice via the regulation of estrogen–progesterone levels in the endometrial epithelium and stroma, and decidual angiogenesis during early pregnancy. Therefore, our findings may provide new evidence for BSHXR as an effective treatment for early pregnancy loss.

The ovarian hormones, estrogen and progesterone, are the key regulators in the implantation and decidualization process. Although the details of these events vary according to different species, the central roles of estrogen and progesterone in the regulation of early uterine events during pregnancy are undoubtable in many mammals (Carson et al., 2000). In COH mice, supraphysiological levels of estrogen and progesterone in early pregnancy were observed, which could lead to implantation defects and improper decidualization (Deng et al., 2013; Ezoe et al., 2014). As discovered in this work, the weight of the ovary and the levels of estrogen and progesterone were significantly increased in COH mice, however, BSHXR could diminish the excessive increased ovarian weight and reduce serum levels of estrogen and progesterone to normal levels. As successful pregnancy primarily depends on the coordination of ovarian hormones and their respective nuclear receptors in the uterus (Cha et al., 2012), we then investigated expression of ERα and PR in the uterus.

There are two isoforms of the estrogen receptor (ER), ERα and ERβ, which are transcribed from distinct genes (Mosselman et al., 1996). Compared with ERβ, ERα is the predominant one mediating the actions of estrogen in the uterus. In ERα-null mice, the growth of the uterus was impaired and blastocyst attachment fails (Lubahn et al., 1993). The ablation of ERα in PR-positive uterine cells led to no decidualization to an artificial stimulus in mutant animals, which indicated a vital role of ERα in decidualization (Ramathal et al., 2010). ERa is expressed in all cell type in the uterus and the deletion of ERα in both epithelial and stromal compartments of the uterus will lead to failure of decidualization, by disturbing the paracrine signals regulated by ERα in both the epithelium and stroma (Pawar et al., 2015). In our study, we found decreased expression of ERα in the epithelium and stroma in COH mice which had poor decidualization. Progesterone could inhibit epithelial proliferation by blocking the paracrine mediators of the mitogenic effects of estrogen, contributing to successful implantation and decreasing ERα expression (Halasz and Szekeres-Bartho, 2013), however, supraphysiological level of progesterone might over-suppress the expression of ERα in both the epithelium and stroma, leading to the defective decidualization in ovarian stimulation mice. BSHXR returned the secretion of progesterone back to normal levels and enhanced the expression of ERα in the uterine epithelium and stroma, which may be beneficial for subsequent decidualization.

Similar to ER, progesterone receptor (PR) exists in at least two isoforms, PRA and PRB, and in contrast to ER, PR isoforms originate from differential promoter usage in a single gene (Ogle, 2002; Jacobsen and Horwitz, 2012). Knockout of both PR isoforms in mice leads to infertility resulting from defective ovarian and uterine function, and these knockout mice display impaired uterine development, ovulation and decidualization (Adams and DeMayo, 2015). The expression of PR is localized in the uterine epithelial and stromal compartments and varies dynamically during implantation and decidualization. Estrogen drives PR expression from the epithelium to the stroma, while progesterone decreases its expression altogether (Wetendorf and DeMayo, 2012). As presented in our study, PR expression was found in the uterine epithelium and stroma of the control group, showing a reduction in epithelial expression as the pregnancy progressed. Since regulation of PR expression by estrogen depends on stromal ER (Large and DeMayo, 2012), the down-regulated expression of stromal ER might lead to less induction of PR expression in the stroma of ovarian stimulation mice. BSHXR could elevate PR expression which raises the responsiveness to progesterone for its pro-fertility effect during pregnancy. Therefore, BSHXR can regulate ERα and PR expression in the uterine epithelium and stroma via the steroid hormones estrogen and progesterone, restoring their intricate balance and interaction during implantation and decidualization for the establishment and maintenance of pregnancy.

The development and maturation of the vascular network is vital for successful placentation and normal embryonic growth, which are modulated by angiogenic factors and circulating hormones. *In vivo*, both estrogen and progesterone stimulate angiogenesis, in particular, estrogen promotes uterine vascular permeability while progesterone stimulates vessel maturation (Demir et al., 2010). However, the supraphysiological level of estrogen and progesterone failed to stimulate vascular density or the development of blood vessels. Since various growth factors are involved in the angiogenic process, vascular endothelial growth factor A (VEGFA), angiopoietin 2 (Ang2) and fibroblast growth factor 2 (FGF2) were further studied in our research.

The VEGF family is one of the most important regulators of angiogenesis, and VEGFA is the predominant isoform in the endometrium (Sugino et al., 2002), regulating uterine angiogenesis in rodents and non-human primates (Walter et al., 2005; Sengupta et al., 2007). A previous study showed that VEGF production is associated with decidualization of stromal cells *in vitro* (Matsui et al., 2004). *In vivo*, mice would abort due to a poor vascular network, resulting from defective expression of VEGF or VEGF receptors, suggesting the crucial role for VEGF in angiogenesis during early pregnancy (Demir et al., 2010). Besides, the angiopoietin family collaborates with the VEGF family to control angiogenesis (Thurston, 2003). In the presence of VEGF, Ang-2 could enable the migration and proliferation of endothelial cells, while without VEGF, Ang-2 destabilizes blood vessels, resulting in vessel regression (Holash et al., 1999). However, we found no significant difference in VEGFA and Ang-2 expression between the four groups in our study.

Fibroblast growth factor family members are very strong inducers of migration and proliferation of endothelial cell *in vitro*, and are also highly angiogenic *in vivo* (Klagsbrun and D’Amore, 1991). Compared with VEGF, FGF2 potently stimulate invasion and the formation of new capillary-like tubes at half the concentration needed by VEGF (Pepper et al., 1991). According to a previous study, FGF2 mRNA is strongly expressed in the mesometrial decidua where extensive angiogenesis takes place (Srivastava et al., 1998). This is consistent with our finding that FGF2 is mainly expressed in the mesometrial decidua during early pregnancy, especially in the VSF area. Prior to the formation of the placenta, VSF functions as the transient blood reservoir for the embryo in emergent conditions. The enlargement and elongation of VSF could be indicative of spiral artery modification and formation of the placenta (Kim et al., 2013). The defective angiogenesis in COH mice, including the insufficient enlargement and elongation of VSF and decreased expression of FGF2 in the VSF area, could lead to pregnancy loss after implantation, and these characteristics could be prevented by BSHXR. In addition to the role of FGF2 in decidual angiogenesis, FGF2 also acts as a paracrine factor related to the mitogenic effects of estrogen on the uterine epithelium (Li et al., 2011). Since estrogen acts through ER in the stroma to induce the secretion of FGFs (Pawar et al., 2014), the decreased expression of ER in the stroma and epithelium may lead to the declined level of FGF2 in COH mice, which then results in deficient decidual angiogenesis. Since BSHXR could enhance ER expression, as well as FGF2 expression, the crosstalk between the stroma and epithelium could be rescued, promoting vascular development and contributing to the wellbeing of the embryo.

However, due to the difference in decidualization and placentation between humans and mice (Malassiné et al., 2003), more clinical evidence needs to be collected and further investigation is needed for the exploration of the mechanisms behind BSHXR’s pro-fertility effects.

## Conclusion

In the present study, we demonstrated that BSHXR could improve implantation, decidualization and decidual angiogenesis in a dose-dependent manner by regulating estrogen and progesterone pathways and FGF2 expression in the uterus, ultimately contributing to better reproductive performance in mice. This study provides some evidences of how BSHXR may benefit patients with early pregnancy loss, and supports the use of BSHXR as a potential therapy for these patients, whether as a herbal prescription in the clinic alone or as an adjunct to assisted reproduction technology.

## Author Contributions

JD and XT are considered as co-first authors, contributing equally to the design of the study, the animal work, the acquisition of data, the analysis and interpretation of the data, and drafting the manuscript. KS, WM, JX, and YS helped the first authors in different steps of experiments. MZ contributed to the study design, experimental setup, supervision of results and final manuscript approval.

## Conflict of Interest Statement

The authors declare that the research was conducted in the absence of any commercial or financial relationships that could be construed as a potential conflict of interest. The reviewer M-CK and handling Editor declared their shared affiliation.
