# Supplementary material for: Bushen Huoxue Recipe Alleviates Implantation Loss in Mice by Enhancing Estrogen–Progesterone Signals and Promoting Decidual Angiogenesis Through FGF2 During Early Pregnancy
Source: Front Pharmacol. 2018 May 15;9:437. doi: 10.3389/fphar.2018.00437 (PMC5962815; doi:10.3389/fphar.2018.00437)
Supplement: Supplementary file 1 [file Image_1.PDF]

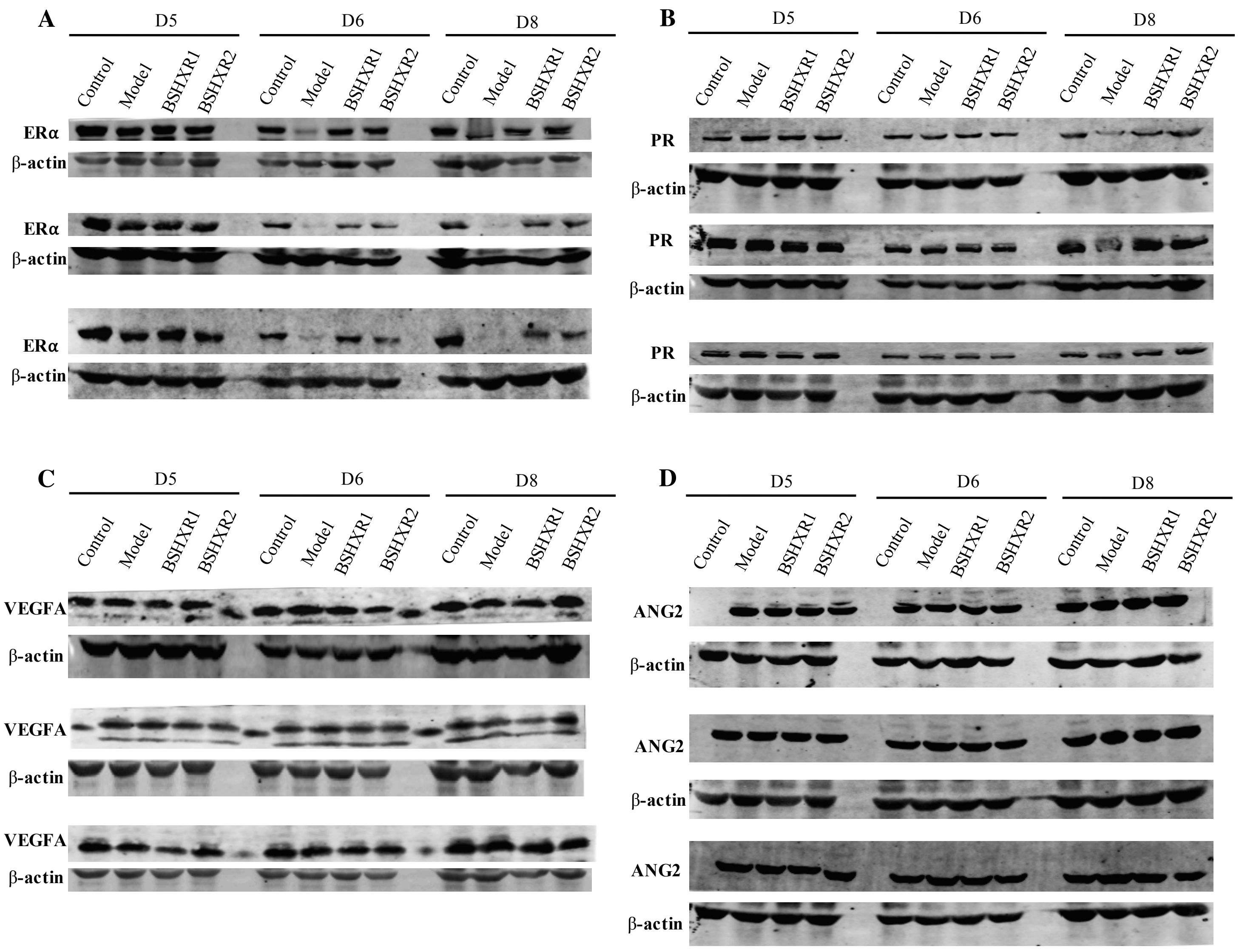

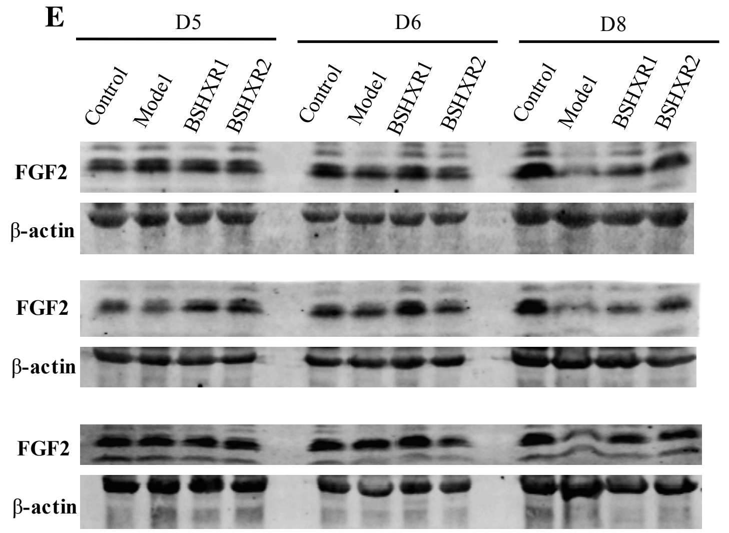

Supplementary Figure 1. Original images from Western blot for ERα (A), PR (B), VEGFA (C), ANG2 (D) and FGF2 (E). N=3.
